# Sensitivity of Bayesian Networks to Noise in Their Parameters

**DOI:** 10.3390/e26110963

**Published:** 2024-11-09

**Authors:** Agnieszka Onisko, Marek J. Druzdzel

**Affiliations:** 1 Faculty of Computer Science, Białystok University of Technology, Wiejska 45A, 15-351 Białystok, Poland; 2 Bayesfusion, LLC, Pittsburgh, PA 15217, USA; marek@bayesfusion.com

**Keywords:** Bayesian networks, numerical parameters, probabilities, noise, sensitivity, medical diagnosis, accuracy

## Abstract

There is a widely spread belief in the Bayesian network (BN) community that the overall accuracy of results of BN inference is not too sensitive to the precision of their parameters. We present the results of several experiments in which we put this belief to a test in the context of medical diagnostic models. We study the deterioration of accuracy under random symmetric noise but also biased noise that represents overconfidence and underconfidence of human experts.Our results demonstrate consistently, across all models studied, that while noise leads to deterioration of accuracy, small amounts of noise have minimal effect on the diagnostic accuracy of BN models. Overconfidence, common among human experts, appears to be safer than symmetric noise and much safer than underconfidence in terms of the resulting accuracy. Noise in medical laboratory results and disease nodes as well as in nodes forming the Markov blanket of the disease nodes has the largest effect on accuracy. In light of these results, knowledge engineers should moderately worry about the overall quality of the numerical parameters of BNs and direct their effort where it is most needed, as indicated by sensitivity analysis.

## 1. Introduction

Decision-analytic methods provide an orderly and coherent framework for modeling and solving decision problems in intelligent systems [1]. A popular modeling tool for complex uncertain domains is a Bayesian network (BN) [2], an acyclic directed graph modeling the structure of a domain and quantified by numerical parameters to represent the joint probability distribution over its variables. BNs are well grounded in probability theory and are thus theoretically sound while at the same time being intuitive. They are the choice tool for those artificial intelligence (AI) problems that require theoretical soundness and reliability. There exist algorithms for reasoning in BNs that calculate the posterior probability distribution over some variables of interest given a set of observations. Due to the fact that these algorithms are mathematically correct, they essentially solve the underlying model. Hence, the ultimate quality of reasoning depends directly on the quality of this model and its parameters. These parameters are rarely precise, as they are often based on subjective estimates. Even if a BN model has been learned from data or is based on hard statistics, it may not be accurate because the data or the statistics may not be directly applicable to the population at hand and not fully trusted [3].

Search for those parameters whose values are critical for the overall quality of decisions is known as sensitivity analysis. Sensitivity analysis studies how much a model output changes as various model parameters vary through the range of their plausible values. It makes it possible to obtain insight into the nature of the problem and its formalization, helps in refining the model so that it is simple and elegant (containing only the factors that matter), and checks the need for precision in refining the numbers [4]. Several researchers proposed efficient algorithms for performing sensitivity analysis in BNs (e.g., [5,6,7,8]).

There is no doubt that it is theoretically possible that small variations in a numerical parameter cause large variations in the posterior probability of interest. Van der Gaag and Renooij [9] demonstrated that typical networks indeed contain such parameters. Due to the fact that practical networks are often constructed with only rough estimates of probabilities, the question of sensitivity of BNs to precision of their parameters is of much interest to builders of intelligent systems. If precision does not matter, rough estimates or even qualitative “order of magnitude” estimates that are typically obtained in the early phases of model building, should be sufficient without the need for their painstaking refinement, perhaps with the exception of some small number of critical parameters. Furthermore, approximate or even fully qualitative schemes might perform well without the need for precise, numerical estimates. Conversely, if BN results are sensitive to the precise values of probabilities, a significant amount of effort needs to be devoted to obtaining precise estimates and it is unlikely that qualitative schemes will match the performance of quantitative problem specifications. There is a popular belief, supported by some anecdotal evidence, that BN models are overall quite tolerant to imprecision in their numerical parameters. Pradhan et al. [10,11] tested this on a very large medical diagnostic model, the CPCS network [12,13]. Their key experiment focused on systematic introduction of noise in the original parameters (assumed to be the gold standard) and measuring the influence of this noise on the average posterior probability of the true diagnosis. They observed that this average was fairly insensitive to even very large noise. This experiment, while ingenious and thought-provoking, had two weaknesses. The first of these, pointed out by Coupé and van der Gaag [7], is that the experiment focused on the average posterior rather than individual posterior in each diagnostic case and how it varies with noise, which is of more interest. The second weakness is that the posterior of the correct diagnosis is by itself not a sufficient measure of model robustness. Practical model performance will depend on how these posteriors are used. In order to make a rational diagnostic decision, for example, one needs to know at least the probabilities of rival hypotheses and, in theory, the joint probability distribution over all disorders. Only then we can weigh the utility of correct against the dis-utility of incorrect diagnosis. If the focus of reasoning is differential diagnosis, it is of importance to observe how the posterior in question compares to the posteriors of competing disorders. Effectively, the question of whether the actual performance of a BN model is robust to imprecision in its numerical parameters remains open. We are not aware of any work that pursued this line of work outside of our work.

In this paper, we present the results of a series of experiments with medical diagnostic BN models. We presented some of these results in specialized workshops [14,15,16] but never in an archival publication. It seems that, while there are citations of this work, there is no follow-up on the subject matter by other researchers. We have amended our experimental setup, extended the set of models tested, conducted additional experiments and are offering a thorough summary of this line of work. In our experiments, we systematically introduce noise in the probabilities and test the diagnostic accuracy of the resulting model. Similarly to Pradhan et al. [10,11], we assume that the original set of parameters and the original model’s accuracy are a gold standard. Adding noise to the original parameters leads to loss of accuracy.

The main result of our analysis is that noise in numerical parameters reduces accuracy not only when it is very large, as suggested by Pradhan et al. Small amounts of noise have minimal effect on accuracy, especially when the noise is biased toward overconfidence, which is typical for parameters obtained from human experts. Given the nature of medical diagnostic models, we also study the impact of noise in each of the four major classes of variables: (1) medical history, (2) physical examination, (3) laboratory tests, and (4) diseases. Although the differences here were rather small, it seemed that noise in the results of laboratory tests and the disease nodes was most influential for the diagnostic accuracy of our models. We also studied the effect of noise in the Markov blanket of the disease nodes, i.e., direct neighborhood of the disease nodes that makes the probabilities of diseases independent of the remainder of the model, and found that parameters in nodes belonging to the Markov blanket have typically larger effect on accuracy than other parameters.

The remainder of this paper is structured as follows. Section 2 provides a brief overview of the datasets (Section 2.1) and models (Section 2.2) used in our experiments. Section 2.3 outlines the architecture of our experiments, Section 2.4 describes our attempt to replicate the results of Pradhan et al. [10,11] and, finally, Section 2.5–Section 2.7 describe our experiments and their results. Finally, Section 3 discusses our results in light of previous work.

## 2. Materials and Methods

Similarly to Pradhan et al. [10,11], for the purpose of our experiments, we assumed that the model parameters were perfectly accurate and, effectively, the diagnostic performance achieved was the best possible. Of course, in reality, a model and its accuracy can be improved upon but this, we believe, does not undermine our results. Any model, no matter how good, can be improved upon. In each of our experiments, we study how this baseline performance degrades as a function of the amount of noise.

We define diagnostic accuracy as the percentage of correct diagnoses on real patient cases. This is obviously a simplification, as one might want to know the sensitivity and specificity data for each of the disorder or look at the global quality of the model in terms of the AUC (Area Under the Curve) measure or the ROC (Receiver Operating Characteristics) curve. This, however, is complicated in case of models focusing on multiple disorders—there is no single measure of performance but rather a measure of performance for every single disorder. We decided thus to focus on the percentage of correct diagnoses, which is a reasonable measure when classes are not too imbalanced.

**Figure 1:**
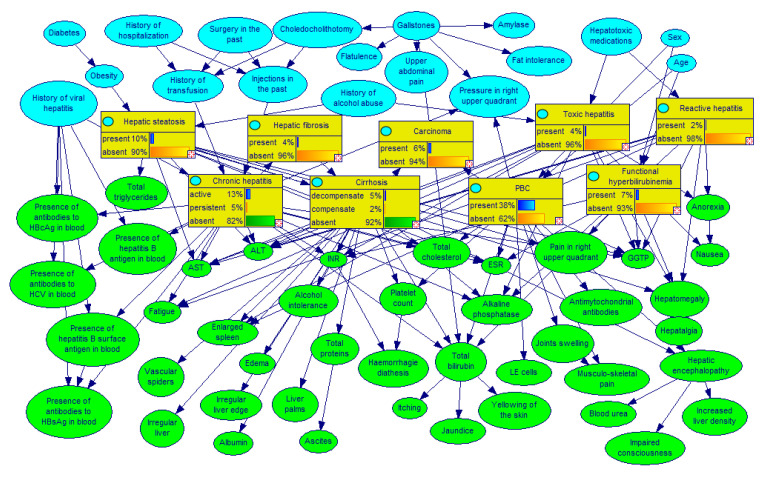
The Hepar II network. Colors represent the role of each node: yellow are disorder nodes, blue are risk factors, history, and demographic data, and green are symptoms, signs, and laboratory tests.

There are a handful of real medical diagnostic Bayesian network models available in Bayesian network repositories and there are several real medical datasets available in machine learning repositories. We have not found a single Bayesian network model that would come with an associated dataset from which it was learned or on which it was tested. This made selecting materials for our experiment daunting. One real and sizeable medical diagnostic model for which we could gain access to real dataset of patient cases is Hepar II, developed by the first author [17]. The Hepar II project aimed at applying decision–theoretic techniques to the problem of diagnosis of liver disorders. Its main component was a BN model involving 70 variables. The model covers 11 different liver diseases (represented by nine separate nodes) and 61 medical findings, such as patient self-reported data, signs, symptoms, and laboratory tests results. The structure of the model, (i.e., the nodes of the graph along with arcs among them) was built based on medical literature and conversations with our domain expert, a Polish hepatologist Dr. Hanna Wasyluk and two American experts, a pathologist, Dr. Daniel Schwartz, and a specialist in infectious diseases, Dr. John N. Dowling. The elicitation of the structure took approximately 50 hours of interviews with the experts, of which roughly 40 hours were spent with Dr. Wasyluk and roughly 10 hours spent with Drs. Schwartz and Dowling. This includes model refinement sessions, where a previously elicited structure was reevaluated in a group setting. Figure 1 shows the structure of the Hepar II network.

An integral part of the Hepar system is its database, created in 1990 and thoroughly maintained since then at the Gastroentorogical Clinic of the Institute of Food and Feeding in Warsaw. The most recent database contains over 800 patient records. Each hepatological case is described by over 160 different medical findings, such as patient self-reported data, results of physical examination, laboratory tests, and a histopathologically verified final diagnosis. The version of the Hepar dataset available to us consisted of 699 patient records. The numerical parameters of the model, i.e., the prior and conditional probability distributions, were learned from the Hepar database. The Hepar II model was tested extensively on data, with experts, and in experiments with general practitioner subjects.

For the purpose of our experiments, we were in dire need of models that came with real sets of patient cases. The task of finding such models is close to impossible. Luckily, BNs can be learned from data (both their structures and their numerical parameters) and most BN software includes learning algorithms. Having access to a real medical dataset allows us thus to build an associated, admittedly imperfect, model. To obtain a collection of models for our experiments, we searched through the public Machine Learning Repository at the University of California Irvine [18] for real medical datasets that are suitable for modeling diagnostic problems. We subsequently built BN models automatically using standard BN learning algorithms available in software GeNIe (version 4.1).

### 2.1. Medical Datasets

We identified seven datasets in the University of California Irvine Machine Learning Repository that were (1) medical datasets, (2) contained at least 100 records, (3) contained at least one class variable (these variables represent the diagnosis, critical for building a diagnostic model), (4) had fewer than five continuous variables (each of these variables had to be discretized and we wanted to minimize the possible bias stemming from discretization), and (5) had fewer than 5% missing values. Standard BN structure learning algorithms cannot handle missing values. We replaced all missing values with states representing absence of a symptom or normal value, which we found in earlier work [17] to lead to highest diagnostic accuracy in medical diagnostic systems. Table 1 summarizes the most important properties of these datasets from the point of view of model building.

As we can see, most of these datasets are rather small and contain a small number of variables. None of them can match the size of the Hepar dataset in terms of the number of variables they contain. Their most important property is that they are real, i.e., describing real patient cases, along with their inherent noise and diagnostic difficulty.

### 2.2. Bayesian Network Models

We used the selected datasets to learn the structures and the parameters of the medical models for our experiments. In each case, we used the entire dataset to derive the structure of an ideal model, a gold standard, that matches the dataset perfectly. In other words, the model represents the joint probability distribution that the data have been drawn from and the records in the dataset fit the learned model precisely/ideally. An anonymous reviewer for *Entropy* suggested that it might be more appropriate to divide the dataset into two parts: (1) data used to learn the model structure, and (2) data used in subsequent experiments. Splitting the dataset into training and testing would reduce the sizes of generally small datasets and we believe that is not really needed in this case. A possible overfitting of the structure to the data makes our gold standard model an even better gold standard model. In that sense, our procedure of learning the structure from the entire dataset is conservative from the point of view of the experimental design. The focus of our experiments is on how the accuracy of this ideal gold standard model deteriorates if there is noise in the parameters. In case of the Hepar II model, the structure was obtained from experts and the problem of obtaining a gold standard structure does not exist—the original Hepar II structure is assumed to be ideal. Regretfully, finding a model like Hepar II and also obtaining access to a dataset of real patient cases is close to impossible.

Table 2 lists all models used in our experiments. Hepar II is the only model that we had ready [17] and did not need to construct. We constructed the remaining models from the datasets listed in Table 1. We used the Bayesian Search [25] structure learning algorithm implemented in GeNIe as the default model learning algorithm, unless a simpler structure, such as TAN (Tree Augmented Naive Bayes) [26] or ANB (Augmented Naive Bayes) [26], led to a better performance. Two models ended up being learned by the TAN and ANB algorithms (Cardiotocography and Breast Cancer, respectively). Table 2 lists the algorithm used in the creation of each model. Subsequent columns give an idea of the complexity of the network in terms of the number of nodes, the average number of states of these nodes, average in-degree (the number of parents of a node), the number of arcs, and the number of numerical parameters. Hepar II is by far the most comprehensive BN in our experiments but the remaining networks offer a reasonable set of models to verify whether our findings are not limited to Hepar II.

**Figure 2:**
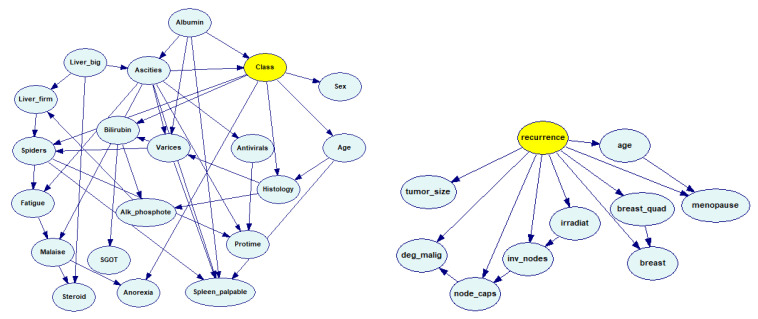
Example BN models learned from data: Hepatitis (**left**) and Breast Cancer (**right**). The yellow nodes (*Class* and *recurrence*) represent class variables.

Figure 2 shows the structure of two models learned from data: Hepatitis and Breast Cancer. The latter was learned using the ANB algorithm, which learns the structure only partially, starting from a Naive Bayes structure and adding connections between features. For this dataset, the ANB-learned structure showed a better diagnostic accuracy than a structure learned by means of the Bayesian Search algorithm.

### 2.3. Experimental Design

To model noise in parameters, we applied the approach proposed by Pradhan et al. [10,11], who introduced noise by transforming each original probability into log-odds form, adding normal noise with a standard deviation σ, and transforming the log-odds form back to probability, i.e.,

(1) $$p^{\prime }=Lo^{-1}\left[Lo\left(p\right)+\mathrm{Normal}\left(0,\sigma \right)\right],$$

where

(2) $$Lo\left(p\right)=\log_{10}\left[p/\left(1-p\right)\right].$$

Pradhan et al.’s experiment involved only binary variables and the transformation always yielded a valid probability. In our case, many model variables contained more than two states. We added a normalization step—after transforming all probabilities within a distribution, we normalized them to make sure that they added up to 1.0.

**Figure 3:**
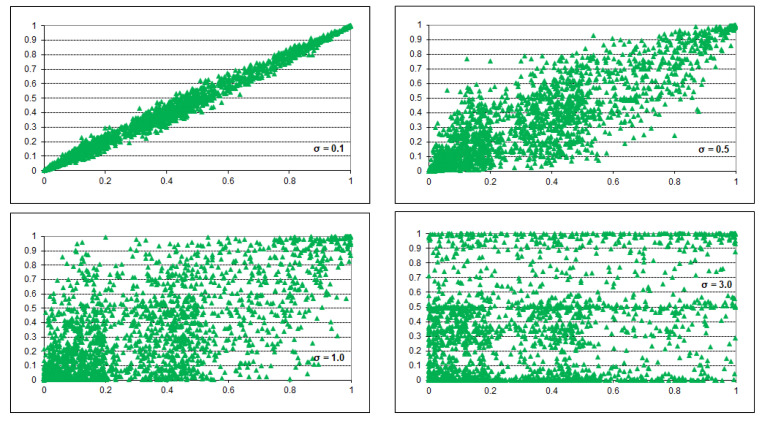
Scatterplots of the original (horizontal axis) vs. transformed (vertical axis) probabilities for the Hepar II model.

The noise introduced using the above method is symmetric in the sense of the value of every parameter changing symmetrically around its original value (see Figure 3, which shows scatterplots of transformed parameters vs. the original parameters for σ=0.1, 0.5, 1.0, and 3.0). We would like to make another observation based on this figure. Introducing noise that goes as far as σ=3.0 does not look realistic, as even for σ=1.0 the points in the plot cover a large plot area and many parameters change drastically under the influence of noise. We decided to limit our experiments to σ∈<0.0,2.0>, which, in our opinion, allows for studying noise that is sufficiently high, perhaps even too high.

In case of parameters obtained from human experts, we may deal with experts who are overconfident or underconfident [27,28]. Modeling overconfidence requires us to make it more likely that the noise drifts toward extreme probabilities (smaller entropy of the probability distribution). Similarly, modeling underconfidence requires the noise to drift toward uniform probabilities (larger entropy of the probability distribution).

We introduced bias into noise in the following way. Given a discrete probability distribution Pr, for overconfidence, we identified the smallest probability $p_{S}$. We transformed this smallest probability $p_{S}$ into $p_{S}^{\prime }$ by making it even smaller, according to the following formula:

(3) $$p_{S}^{\prime }=Lo^{-1}[Lo\left(p_{S}\right)-|\mathrm{Normal}\left(0,\sigma \right)\left|\right].$$

We made the largest probability in the probability distribution Pr, $p_{L}$, larger by precisely the amount by which we decreased $p_{S}$, i.e.,

$$p_{L}^{\prime }=p_{L}+p_{S}-p_{S}^{\prime }.$$

For underconfidence, we identified the largest probability $p_{L}$. We then transformed $p_{L}$ into $p_{L}^{\prime }$ by making it smaller, according to the following formula:

(4) $$p_{L}^{\prime }=Lo^{-1}[Lo\left(p_{L}\right)-|\mathrm{Normal}\left(0,\sigma \right)\left|\right].$$

We made the smallest probability in the probability distribution Pr, $p_{S}$, higher by precisely the amount by which we decreased $p_{L}$, i.e.,

$$p_{S}^{\prime }=p_{S}+p_{L}-p_{L}^{\prime }.$$

We were by this guaranteed that the transformed parameters of the probability distribution ${\mathrm{Pr}}^{\prime }$ added up to 1.0.

**Figure 4:**
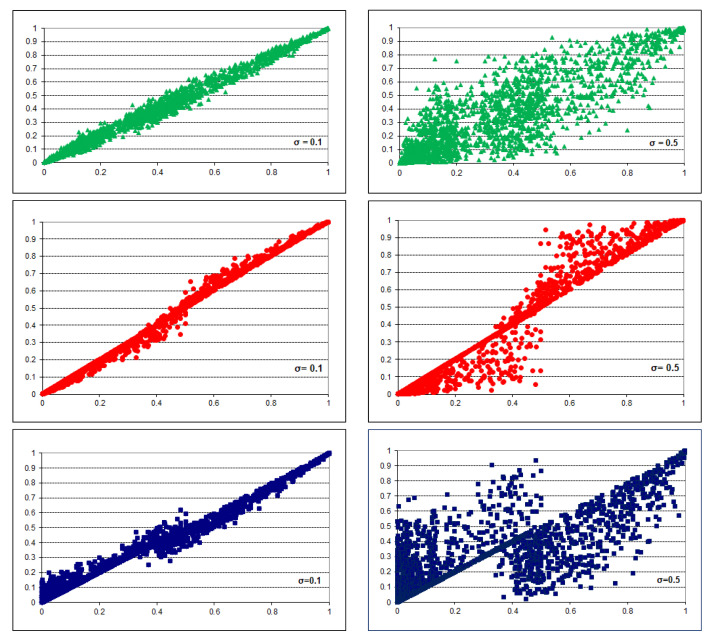
Scatterplots of the original (horizontal axis) vs. transformed (vertical axis) probabilities for σ=0.1 and σ=0.5. The top two plots show symmetric noise, the middle two plots show overconfidence, the bottom two plots show underconfidence.

To give the reader an idea of the effect of the transformations, we show in Figure 4 scatterplots of the transformed probabilities as a function of their original values for the three types of noise: symmetric (top row), overconfidence (middle row), and underconfidence (bottom row) for two values of σ: 0.1 and 0.5. It is clear from the scatterplots that higher values of σ lead to more noise in the parameters. Value σ=0 would result in a diagonal line on the plot. Value σ=0.1 leads to a slight distortion of the parameters while distortion for σ=0.5 is significant: some parameters change their values from, for example, 0.1 to 0.8. Biased noise makes the parameters more (overconfidence) or less (underconfidence) extreme. Extreme probabilities, characterized by a small entropy of the underlying probability distribution, express higher confidence of the source of the probabilities in the knowledge of the variables and their interactions. An expert who expresses probabilities that are close to 0.0 or 1.0 is quite certain of his or her knowledge.

In each experiment described in the following sections, we use leave-one-out cross-validation while measuring the diagnostic accuracy of our models. For every data record in a dataset of *n* records, we learn the parameters of the gold standard model from the remaining *n* − 1 records, mutilate these parameters by introducing noise, and test the mutilated model on the one record that we have set aside. Accuracy is the number of correctly guessed class taken over all *n* records in the leave-one-out procedure. Introducing noise with a given value of sigma during each iteration of the leave-one-out procedure controls the variance in the results.

### 2.4. Replication of the Experiment of Pradhan et al.

As a starting point, we replicated the experiment performed by Pradhan et al. [10,11] on the Hepar II model. The original experiment focused on the robustness of the posterior probability of each disease to noise in the context of a very large medical diagnostic network, CPCS [12,13]. They transformed the CPCS’ parameters by what we called in Section 2.3 *symmetric noise* with σ∈<0.0,3.0> and found that the posterior probability of the true diagnosis changed little with rising values of σ, concluding that noise had limited impact on the accuracy of the model.

Hepar II models 11 diseases of the liver. Similarly to Pradhan et al., we derived the posterior probability assigned by the Hepar II network to the true diagnosis, averaged over the set of all test cases for σ∈<0.0,3.0> with 0.1 increments.

**Figure 5:**
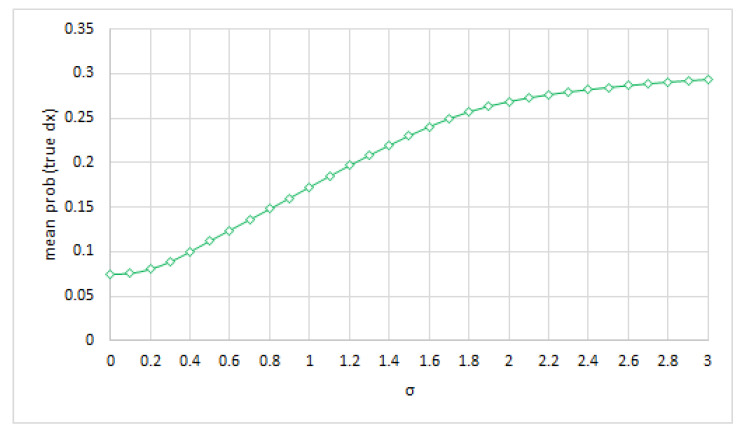
The average posteriors for the true diagnoses as a function of unbiased (symmetric) noise.

The results, shown in Figure 5, seem to indicate, similarly to Pradhan et al., that the average posteriors are not sensitive to accuracy in probabilities. These posteriors actually increased with the increase in σ. In the context of the Hepar II model this is quite likely due to an overall increase in a priori probabilities of all diseases. The prevalence of each of the disease is rather small and symmetric noise typically increases it. Please note that Hepar II is a multiple-disorder model.

**Figure 6:**
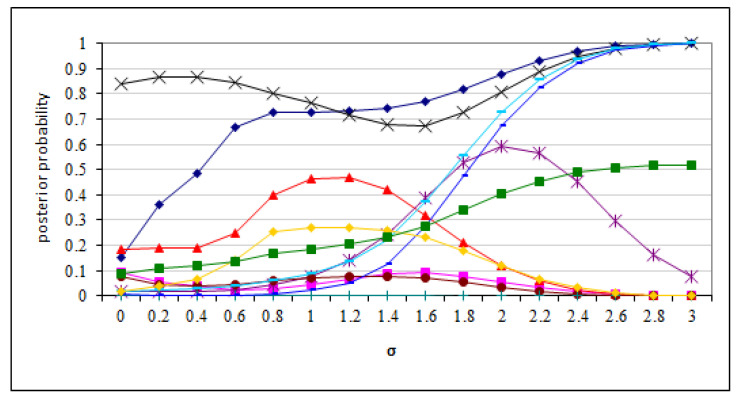
The posterior probabilities of Hepar II disorders as a function of σ on a single patient case. The lines represent posterior probabilities of the 11 disorders.

To probe this result deeper, we focused on how individual posteriors of each of the disorders included in the model change as a function of noise for a handful of randomly selected patient cases. Here we observed that not only the probabilities, but also the order between the probabilities of various diseases changes, demonstrating that average over all runs does not reflect sensitivity well. Figure 6 shows the plot of posterior probabilities of the 11 disorders as a function of noise on a single selected patient case. This case is representative for the cases that we examined. We can see that the posterior probabilities change with the noise to the point of changing the order of the most probable diagnoses. This plot illustrates the two weaknesses of Pradhan et al.’s experiment listed in the Introduction (Section 1). The demonstrated weaknesses question the adequacy of the measure applied by Pradhan et al. It seems that the average posterior of the correct diagnosis stops short of measuring the actual impact of noise on the diagnostic accuracy.

### 2.5. Experiment 1: Effect of Noise on Models’ Diagnostic Accuracy

Our first experiment focused on the diagnostic accuracy of each of the eight models listed in Table 2 for each of the three types of noise described in the previous section. We tested each of the five versions of each network (each for a different standard deviation of the noise σ∈<0.0,2.0> with 0.5 increments) on the set of test cases (the original dataset for each BN model) and computed its diagnostic accuracy.

**Figure 7:**
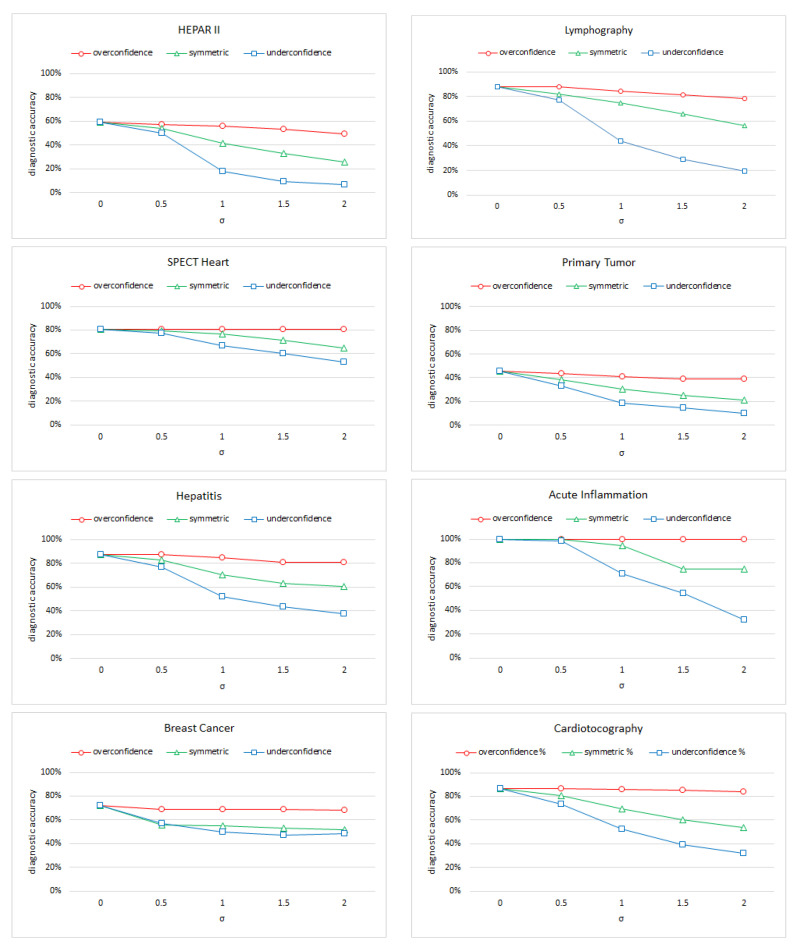
The diagnostic accuracy of the eight models (clock-wise: Hepar II, Lymphography, Primary Tumor, Acute Inflammation, Cardiotocography, Breast Cancer, Hepatitis, and Spect Heart) as a function of the amount of unbiased (symmetric) and biased (overconfidence and underconfidence) noise.

Figure 7 shows the diagnostic accuracy of each of the eight models (clock-wise: Hepar II, Lymphography, Primary Tumor, Acute Inflammation, Cardiotocography Breast Cancer, Hepatitis, and Spect Heart) as a function of the amount of unbiased (symmetric) and biased (overconfidence and underconfidence) noise. Line colors correspond to the colors of the scatterplots in Figure 4.

We can see that the accuracy of each of the models decreases with the amount of noise, although the effect is different for unbiased (symmetric) and biased noise. Overconfidence leads to the smallest decrease in accuracy, followed by unbiased (symmetric) noise and underconfidence. The decrease in accuracy for overconfident noise is minimal. It seems that overconfident noise strengthens the models’ ability to diagnose correctly. This result is consistent across all BN models studied. Another observation that seems qualitatively consistent across all models is that a small amount of noise (say, σ<0.2) does not impact the accuracy of the model significantly. The gold standard Acute Inflammation model is 100% accurate. We have browsed through a handful of papers citing the *Acute Inflammation* dataset and noticed that most machine learning/classification methods applied by other authors give accuracy close to 100%, which means that the dataset is rather easy and we have hit the ceiling effect here.

### 2.6. Experiment 2: Symmetric Noise in Various Diagnostic Types of Nodes

Given a medical diagnostic model, it is of interest to know which of the semantically distinct parts of the model (i.e., medical history, physical examination, disease nodes, and laboratory results) has the highest influence on model’s diagnostic accuracy. We addressed this question by introducing noise in these parts of the model only and measuring the model’s diagnostic accuracy. Unfortunately, we were able to identify the diagnostic node types only in case of the Hepar II network, which the first author had constructed with help of medical experts. Identification of variables that denote medical history, physical examination, and laboratory results would require considerable amount of guesswork in case of the datasets used in our experiments.

**Figure 8:**
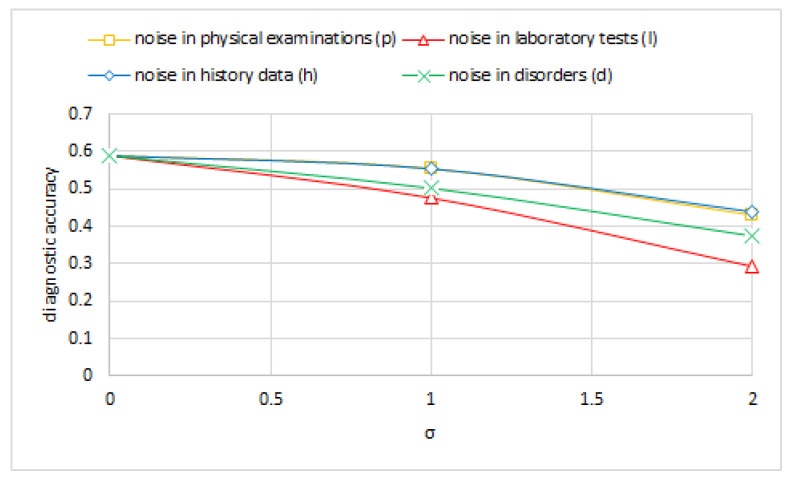
The diagnostic accuracy of various semantic parts (physical examinations, laboratory results, history, and disorders) of the Hepar II model as a function of the amount of unbiased (symmetric) noise.

We tested each of the three versions of the Hepar II network (each for a different standard deviation of the noise σ∈<0.0,2.0> with 1.0 increments) on the set of test cases and computed its diagnostic accuracy. Figure 8 shows the diagnostic accuracy of the model for noise in each part of the network as a function of σ. In this figure, *h* represents the version of the network with noise introduced only to history variables, i.e., those that represent data acquired during an interview with a patient. Similarly, *p* stands for physical examination, *l* for laboratory tests, and *d* for disorders. We can observe that noise in the results of laboratory tests impacts the diagnostic performance of our model most. This is not really surprising, as laboratory tests are highly diagnostic compared to history data and results of physical examination, which are to a large degree subjective. Noise in the disease nodes is also important.

### 2.7. Experiment 3: Noise in Nodes Belonging to the Markov Blanket of the Disorder Nodes

**Figure 9:**
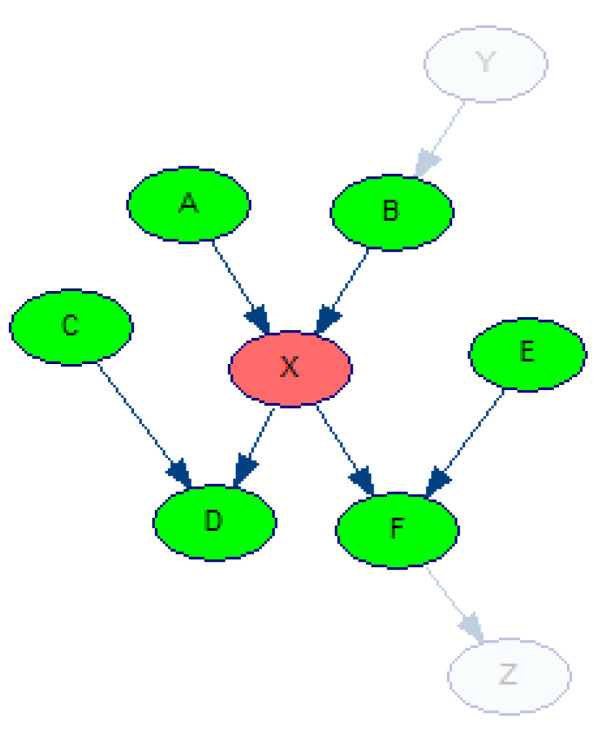
An example of a Bayesian network model with a Markov blanket of the node *X*. The nodes in green depict the Markov blanket of the node in red.

The Markov blanket of a node *X* is the set of nodes including: (1) parent nodes of *X*, (2) children nodes of *X*, and (3) parent nodes of children nodes of *X*. Figure 9 shows an example BN with the Markov blanket of the node *X* highlighted in green (nodes *A*, *B*, *C*, *D*, *E*, and *F*).

There is an important relationship between a node *X* and nodes that are not part of *X*’s Markov blanket. This relationship is described by Equation (5), which shows that the node *X* is conditionally independent of nodes outside its Markov blanket (nodes *Y* and *Z* in Figure 9) given the nodes that belong to its Markov blanket (MB(X) = {*A*, *B*, *C*, *D*, *E*, *F*} in Figure 9):

(5) P(X|MB(X),Y,Z)=P(X|MB(X)).

**Figure 10:**
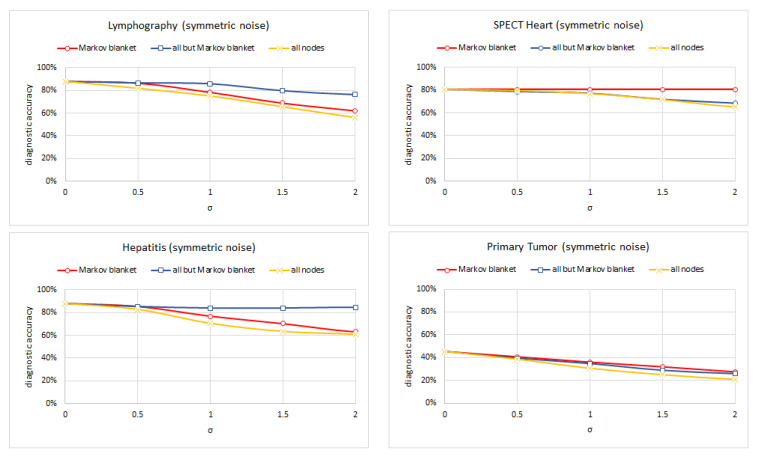
The diagnostic accuracy of the four models (clock-wise: Lymphography, Spect Heart, Primary Tumor, and Hepatitis) as a function of the amount of unbiased (symmetric) noise.

**Figure 11:**
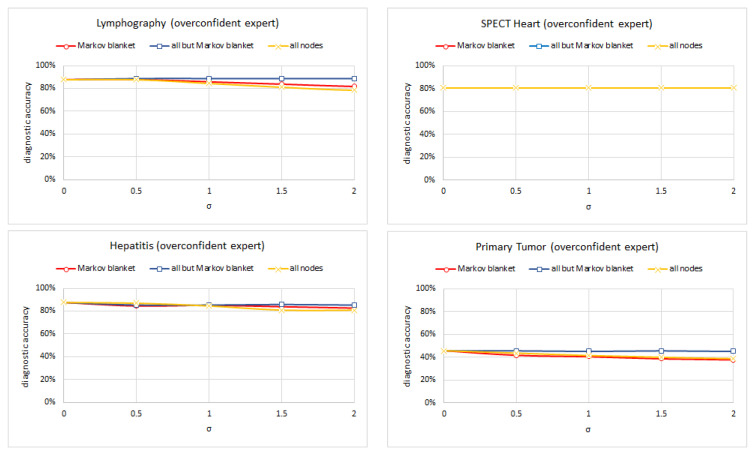
The diagnostic accuracy of the four models (clock-wise: Lymphography, Spect Heart, Primary Tumor, and Hepatitis) as a function of the amount of biased noise (overconfidence).

**Figure 12:**
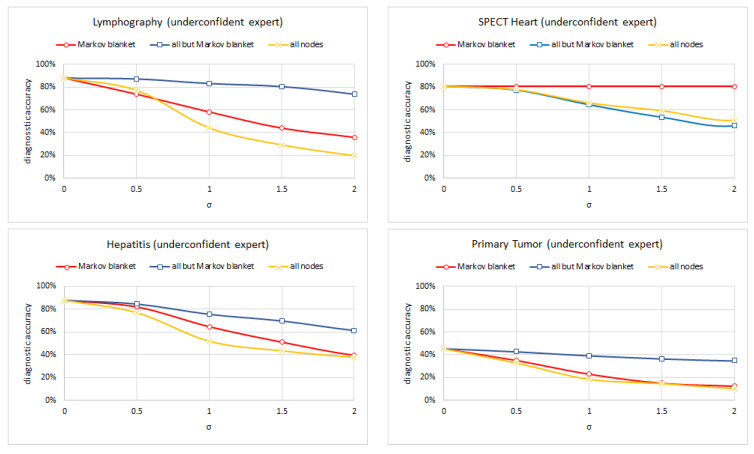
The diagnostic accuracy of the four models (clock-wise: Lymphography, Spect Heart, Primary Tumor, and Hepatitis) as a function of the amount of biased (underconfidence).

Experiment 3 consisted of testing each of five versions of the following four models: (Lymphography, Spect Heart, Hepatitis, and Primary Tumor) (each for a different standard deviation of the noise σ∈<0.0,2.0> with 0.5 increments) on the set of test cases and computed its diagnostic accuracy. We left out the Acute Inflammation, Cardiotocography, and Breast Cancer models because their structure made the Markov blanket trivial. In case of the models learned using the TAN and ABN algorithm, all nodes belong to the Markov blanket of the class node. Hepar II and Acute inflammation models had multiple disease nodes and the set sum of the Markov blankets included all nodes. Figure 10, Figure 11 and Figure 12 show the results of this experiment for symmetric, overconfidence, and underconfidence noise, respectively. Each figure presents three lines corresponding to three models (1) a model with noise introduced into the Markov blanket nodes (red line); (2) a model with noise introduced to all but Markov blanket nodes (blue line); and (3) a model with noise introduced into all nodes (yellow line). Please note that in Figure 11, the top-right chart shows only one line. This is the result of the three lines being almost identical and covering each other. Table 3 summarizes the properties of the Markov blankets of the selected models.

We can see that noise in the Markov blanket of the disease nodes is important and typically impacts the diagnostic accuracy most. The differences are minimal for the overconfident noise, as the accuracy decreases slowly in this case (this is consistent with the results of Experiment 1). The results for the Spect Heart model differ here but are quite likely the result of the Markov blanket containing only three nodes. Noise in only three nodes does not impact the diagnostic accuracy too much.

## 3. Discussion

The main result of our analysis is that noise in numerical parameters reduces accuracy not only when it is very large, as suggested by Pradhan et al. Small amounts of noise have minimal effect on accuracy, especially when the noise is biased toward overconfidence, which is typical for parameters obtained from human experts. We believe that there are two possible explanations of this discrepancy in results. The first and foremost is that Pradhan et al. used a different criteria for model performance—the average posterior probability of the correct diagnosis. We pointed out problems with this measure in Section 1 and concluded in Section 2.4 that it may not reflect well the practical performance of BN models, focusing our work on the diagnostic accuracy of the model. Another, although we believe that it is a less influential factor, may be differences between the models under study. The CPCS network used by Pradhan et al. consisted solely of Noisy-OR gates [29], which may behave differently than general nodes. In Hepar II, only roughly 50% of all nodes could be approximated by Noisy-MAX [30] and the remaining models, learned from real medical datasets, contained no Noisy-OR nodes.

Overconfidence bias had, in our experiments, a smaller negative effect on model performance than symmetric noise. Underconfidence bias led to the most serious deterioration in performance. While it is only a wild speculation that begs for further investigation, one might see our results as an explanation why humans have developed to be overconfident rather than underconfident in their probability estimates.

We also studied the influence of noise in each of the four major classes of variables: (1) medical history, (2) physical examination, (3) laboratory tests, and (4) diseases, on the diagnostic performance (Experiment 2). It seemed that noise in the results of laboratory tests was most influential for the diagnostic performance of our models. This can be explained by the high diagnostic value of laboratory tests and the decrease in this value with the introduction of noise.

Finally, we looked at the structural considerations in testing the impact of the structure on the effect of noise by introducing noise into the Markov blanket of the disease nodes (Experiment 3). Noise in the Markov blanket of the disease nodes, i.e., nodes that were topologically closes to the disease nodes, seemed to have the largest impact on the overall accuracy of the model, although not always, which we think could be just a result of random anomalies. SPECT Heart model shows a minimal effect of noise in the Markov blanket of the class node. This could be because the Markov blanket of the class node consists of only three nodes. In case of symmetric noise in the Primary Tumor model, the effect of noise in the Markov blanket is smallest but very similar to the effect of noise in other nodes.

The results of our experiment touch upon the foundations of qualitative modeling techniques. As qualitative schemes base their results on approximate or abstracted measures, one might ask whether their performance will match that of quantitative schemes, either in terms of their strength or the correctness of their results.

While our result is merely a single data point that sheds light on the hypothesis in question, we suggest that BNs may be more sensitive to the quality of their numerical parameters than popularly believed, although we have observed that small amounts of noise do not impact the overall accuracy too much. We argue that further empirical studies of this topic should use hard context-dependent performance measures (such as the quality or correctness of system’s recommendation). Proxy variables, such as the average posterior probabilities of correct diagnosis, may not be adequate to evaluate the robustness of a model. Alternatively, one might use measures such as admissible deviation (a change in probability that does not impact the order of most likely diagnoses) proposed by van der Gaag and Renooij [9].

We have conducted a related study of the impact of imprecision (rather than noise that we are focusing on in this paper) in BN parameters on the accuracy of BNs [31]. In that study, we progressively rounded the parameters instead of transforming them with noise and observed minimal impact of imprecision on model accuracy. That work has so far (2024) seen almost a hundred citations. While there are citations to the line of work described in the current paper, there seems to be no follow-up in terms of replicating our studies or proposing an alternative way of addressing the question of sensitivity of BNs to noise. We expect that the current paper will spark renewed interest in this important issue.

## Figures and Tables

**Table 1:** Medical datasets used in our experiments.

Data Set	Citation	Instances	Variables	Variable Types	Classes	Missing Values
Hepar	—	699	70	categorical, real	11	yes
SPECT Heart	[19]	267	23	categorical	2	no
Lymphography	[20]	148	19	categorical, integer	4	no
Hepatitis	[21]	155	20	categorical, real	2	yes
Primary Tumor	[20]	339	18	categorical, integer	20	yes
Acute Inflammation	[22]	120	8	categorical, integer	4	no
Cardiotocography	[23]	2126	22	categorical, real	3	no
Breast Cancer	[24]	286	10	categorical	2	yes

**Table 2:** Bayesian network models used in our experiments.

Model	Structure Learning Algorithm	Number of Nodes	Average Number of States	Mean in-Degree	Number of Arcs	Number of Parameters
Hepar II	—	70	2.31	1.76	123	2139
SPECT Heart	BSA	23	2.00	2.26	52	290
Lymphography	BSA	19	3.00	1.05	20	300
Hepatitis	BSA	20	2.50	1.90	38	465
Primary Tumor	BSA	18	3.17	1.83	33	877
Acute Inflammation	BSA	8	2.13	1.88	15	97
Cardiotocography	TAN	22	2.91	1.86	41	534
Breast Cancer	ANB	10	4.50	1.40	14	200

**Table 3:** Number of nodes in Markov blanket.

Model	Number of Nodes	Number of Nodes in Markov Blanket (%)
SPECT Heart	23	3 (13%)
Lymphography	19	9 (47%)
Hepatitis	20	11 (55%)
Primary Tumor	18	11 (61%)

## Data Availability

All datasets described in this paper, except for the Hepar data, which are proprietary, are openly available in the University of California Irvine Machine Learning Repository at https://archive.ics.uci.edu/ (accessed on 1 September 2024). Hepar II model is available at the BayesFusion, LLC’s, interactive model repository at https://repo.bayesfusion.com/ (accessed on 1 September 2024).

## Abbreviations

The following abbreviations are used in this manuscript:

ANB	Augmented Naive Bayes (structure learning algorithm)
AI	Artificial Intelligence
BN	Bayesian Network
BSA	Bayesian Search (structure learning algorithm)
TAN	Tree Augmented Naive Bayes (structure learning algorithm)
